# Safety of Selective and Routine Femoral Access Angiography Post-Transcatheter Aortic Valve Implantation

**DOI:** 10.1016/j.jacadv.2026.103127

**Published:** 2026-08-14

**Authors:** Alexander von Ehr, Jonathan Rilinger, Derek Hazard, Tasnim Mafiz, Bui Bao Khanh Dinh, Vera Oettinger, Thomas Mauhardt, Lukas A. Heger, Christoph B. Olivier, Alicja Zientara, Roman Gottardi, Martin Czerny, Ingo Hilgendorf, Uwe Zeymer, Dirk Westermann, Constantin von zur Mühlen, Alexander Maier

**Affiliations:** a Department of Cardiology and Angiology, University Heart Center Freiburg-Bad Krozingen, Faculty of Medicine, University of Freiburg, Freiburg, Germany; b DZHK (German Centre for Cardiovascular Research), Berlin, Germany; c Department of Cardiology, Angiology and Intensive Care Medicine, Deutsches Herzzentrum der Charité (DHZC), Campus Virchow Klinikum, Berlin, Germany; d Institute of Medical Biometry and Statistics, Faculty of Medicine and Medical Center, University of Freiburg, Freiburg, Germany; e Department of Cardiovascular Surgery, University Heart Center Freiburg-Bad Krozingen, Faculty of Medicine, University of Freiburg, Freiburg, Germany

**Keywords:** access site, bleeding, iliofemoral angiography, TAVI, VARC-3, vascular complications

## Abstract

**Background:**

Postprocedural iliofemoral angiography is commonly used to detect access-site complications after transfemoral transcatheter aortic valve implantation (TF-TAVI). However, the clinical value of routine angiographic surveillance remains uncertain.

**Aims:**

To compare vascular and access-site-related complications according to Vascular Academic Research Consortium-3 (VARC-3) criteria associated with routine and selective postprocedural iliofemoral angiography after TF-TAVI.

**Methods:**

We retrospectively analyzed 1,147 TF-TAVI procedures at 2 sites of a single center with different institutional strategies for postprocedural angiographic assessment. One site routinely performed iliofemoral angiography after TAVI (n = 674), whereas the other applied a selective strategy based on procedural suspicion (n = 473).

**Results:**

Vascular and access-site complications (VARC-3) were similar between strategies (selective 6.6% vs routine 8.0%; OR: 0.76; 95% CI: 0.48-1.19). Routine angiography detected more access-site bleedings (13.9% vs 5.2%; *P* < 0.001), without differences in hemoglobin drop, transfusion due to access-site bleeding, or bleeding-related death. Overall vascular reinterventions were comparable (4.2% vs 6.5%; *P* = 0.095), with more catheter-based interventions in the routine strategy and more surgical/thrombin-based interventions in the selective strategy. In adjusted and inverse probability of treatment weights–weighted analyses, bleeding complications remained lower with the selective strategy, whereas overall VARC-3 complications did not differ. After adjustment using the inverse probability of treatment weights model, the angiography strategy was not associated with acute kidney injury despite higher total contrast in the routine group.

**Conclusion:**

Routine angiography increases detection of minor vascular abnormalities, while selective angiography guided by clinical suspicion may provide comparable clinical outcomes. Routine postprocedural angiography may be unnecessary in many patients. Prospective randomized studies are needed to determine the optimal surveillance strategy.

Transfemoral transcatheter aortic valve replacement (TF-TAVI) is a cornerstone treatment for patients with severe aortic stenosis, particularly in elderly individuals or those deemed high risk for conventional surgical aortic valve replacement.^1^, ^2^, ^3^, ^4^ As procedural techniques and device technologies have advanced, the safety profile of TAVI has been markedly improved.^5^ Nonetheless, vascular access-related complications remain among the most frequent adverse events given the reliance on transfemoral arterial access in the majority of cases.

Vascular complications following TAVI—such as access-site bleeding, arterial dissection, perforation, or occlusion—can lead to significant morbidity, prolonged hospitalization, and increased mortality.^6^ Consequently, accurate and timely detection of these events is critical for optimizing patient outcomes. One often employed strategy to identify such complications involves postprocedural angiographic assessment of the iliofemoral arteries.^7^^,^^8^ This imaging is typically performed immediately after valve implantation via the secondary access, allowing for the detection of clinically silent but potentially relevant access-site complications.

A substantial proportion of TAVI candidates present with concomitant peripheral artery disease (PAD), particularly affecting the iliofemoral and femoropopliteal segments. These patients are at heightened risk of vascular complications due to pre-existing vessel stenoses, calcifications, tortuosity, and reduced arterial compliance.^9^ In such cases, even minimal mechanical trauma or suboptimal access technique can precipitate clinically significant vascular injury.^10^^,^^11^ The presence of PAD not only complicates vascular access and closure but may also obscure early clinical signs of access-site compromise, thereby increasing the potential benefit of systematic imaging-based surveillance. Despite these considerations, there is currently no universal consensus on the necessity or frequency of routine post-TAVI iliofemoral angiography. Clinical practice varies between centers and among operators, with most institutions adopting a standardized protocol involving angiography or even digital subtraction angiography in all patients, while some rely on a more selective, indication-driven approach based on clinical signs or procedural suspicion.

Whether a systematic approach to postprocedural angiography leads to improved detection and management of vascular complications—and thereby translates into better clinical outcomes—remains unclear. The present study aims to evaluate the impact of routine vs selective iliofemoral angiographic assessment following TAVI on the incidence of vascular complications and related clinical outcomes.

## Methods

This study followed the Reporting of studies Conducted using Observational Routinely-collected health Data (RECORD) reporting guidelines.^12^

### Study population

The population of this study is based on the University Heart Center Freiburg–Bad Krozingen TAVI registry, which analyzes retrospectively and recruits prospectively patients from both sites of the center. Both retrospective and prospective TAVI cohorts were included in the analysis. All consecutive patients who underwent TAVI at the University Heart Center Freiburg–Bad Krozingen between February 2020 and June 2022 were retrospectively included into the study (retrospective cohort; “Transcatheter Aortic Valve Implantation Registry of the University Heart Center Freiburg–Bad Krozingen,” DRKS-ID: DRKS00029242). Subsequently, from June 2022 to February 2024, all patients with a heart team–based indication for TAVI due to severe symptomatic aortic stenosis—according to the 2021 European Society of Cardiology/European Association of Cardio-Thoracic Surgery guidelines—were prospectively enrolled at the Freiburg site and included into this analysis (DRKS-ID: DRKS00034681).

The retrospective and prospective study protocols were approved by the local ethics committee (EK-Freiburg 22-1453-S1; 21-1358). Written informed consent was obtained from all patients in the prospective cohort. For the retrospective cohort, the ethics committee waived the requirement for written informed consent. Operators were not instructed about this study’s hypothesis, and data was collected blinded to this analytic objective.

Patients with transapical or subclavian TAVI were excluded from this analysis.

Primary access-site characteristics (primary access kinking and calcification) were collected on a routine basis in the registry. The detailed evaluation method is described elsewhere.^13^

### Study design and angiography protocols

TF-TAVI was conducted at the 2 sites of the University Heart Center Freiburg–Bad Krozingen. Both are high-volume TAVI sites with a standardized transfemoral approach. Site protocols differed with regard to the use of postprocedural iliofemoral angiography for the detection of primary vascular access complications. At the Bad Krozingen site, a routine angiographic assessment of the iliofemoral vasculature was systematically performed at the end of each TAVI procedure. In contrast, at the Freiburg site, postprocedural angiography was performed at the discretion of the implanting physician. These indications were not strictly defined but comprised persistent access-site bleeding after closure, expanding hematoma, loss or weakening of distal pulses, unexplained hypotension, abnormalities during sheath removal, or difficult vascular closure. In the absence of such indicators, no routine imaging was performed at the Freiburg site.

Postprocedural bleeding and vascular complications were defined according to the Valve Academic Research Consortium-3 (VARC-3) criteria.^14^ Primary outcomes were major and minor vascular and access-related complications according to VARC-3, occurrence of bleeding (composed of access-site bleeding = any access-site type 1-4 bleeding according to VARC-3 criteria), closure device failure (major or minor according to VARC-3 vascular and access-related complications), hemoglobin drop >3 g/dL (VARC-3 type 2 or greater bleeding), bleeding-related death according to VARC-3, vascular complications (composed of dissection, perforation, stenosis, occlusion, pseudoaneurysm), and the necessity of vascular reintervention post TAVI (composed of surgical repair, catheter-based intervention, thrombin injection).

Secondary outcomes included fluoroscopy time, blood transfusion, timing of vascular reintervention (periprocedural, postprocedural <48 hours, postprocedural >48 hours), and acute kidney injury (defined as increase of serum creatinine by ≥0.3 mg/dL within 48 hours or increase in serum creatinine to ≥1.5 times baseline following Kidney Disease: Improving Global Outcomes [KDIGO] guidelines).^15^

### Postprocedural iliofemoral angiographic assessment

After successful valve deployment, the primary femoral TAVI access sheath was removed and sealed by vascular closure devices. The primary access standard closure procedure at both the Freiburg and Bad Krozingen sites is a primary suture-based vascular closure strategy (ProGlide, Abbott Vascular). Two devices were inserted before the procedure and deployed at the 10-o’ and 2-o’clock positions. After the procedure was concluded, the introducer sheath was removed, and the suture-based technique was executed with a safety wire in place for the use of additional devices. If needed, an additional small plug-based closure device (Angioseal, Terumo) was used. In case of persistent bleeding, a large plug-based closure device was employed (MANTA, Teleflex).

Primary access angiography was performed via the secondary access using a pigtail or multipurpose diagnostic catheter. A nonselective angiography of the pelvic and femoral arteries was conducted by administering 15 to 25 mL of iodinated contrast medium at a rate of 10 to 15 mL/s. The imaging field included the external iliac, common femoral, and superficial femoral arteries to allow for complete visualization of the access site and proximal vessel segments.

All angiographic images were evaluated in real time by the interventional cardiologist performing the procedure. Findings suggestive of vascular injury—such as dissections, perforations, flow-limiting stenoses, pseudoaneurysms, or contrast extravasation—were documented and, if necessary, immediately managed with endovascular techniques (eg, prolonged balloon inflation, stent placement, or surgical consultation).

### Vascular access technique and periprocedural medication

Femoral arterial access was obtained according to institutional standards at both sites. Ultrasound guidance was routinely used for puncture in the common femoral artery. The level of the femoral bifurcation was assessed using a routine computed tomography angiogram, and the puncture site was selected accordingly.

Periprocedural antithrombotic therapy followed contemporary guideline recommendations and institutional protocols. Patients undergoing TAVI received intraprocedural unfractionated heparin targeting an activated clotting time above 250 seconds. Antiplatelet or anticoagulant therapy at the time of the procedure was administered according to the clinical indication (eg, atrial fibrillation, prior coronary stenting). Oral anticoagulation was paused for the procedure.

### Statistical analysis

Continuous variables are presented as mean ± standard deviation or median with interquartile range, as appropriate. Categorical variables are expressed as absolute frequencies and percentages. A complete case analysis was performed. Binominal logistic regression was used for a univariable analysis of primary and secondary outcomes. To account for potential confounding factors, binominal multivariable logistic regression analyses were performed for the 4 primary outcomes (vascular and access-related complications according VARC-3, bleeding complications, vascular complications, and vascular re-interventions) and the secondary outcomes with sufficient event counts for calculation. Covariates for multivariable adjustment (post-TAVI femoral angiography strategy, age, sex, body mass index [BMI], Society of Thoracic Surgeons [STS] score, hypertension, diabetes, hyperlipidemia, smoking status, ballon-expandable prosthesis, prosthesis diameter, PAD) were selected a priori based on clinical relevance. Model calibration was assessed using the Hosmer–Lemeshow goodness-of-fit test.

In addition, as a sensitivity analysis, propensity score weighting was performed to account for baseline differences between the participants undergoing the procedures. A logistic regression model estimated the probability of receiving routine vs selective postprocedural angiography using clinically relevant baseline covariates (age, sex, BMI, STS score, hypertension, diabetes, hyperlipidemia, smoking status, balloon-expandable prosthesis, prosthesis diameter, PAD). Stabilized inverse probability of treatment weights (IPTW) were calculated to estimate the average treatment effect. To reduce the influence of the extreme weights and improve model stability, weights were truncated at the 1st and 99th percentiles. Covariate balance was assessed using absolute standardized mean differences (SMD < 0.10 indicated adequate balance). IPTW-weighted logistic regression models were fitted for primary and secondary outcomes.

Adjusted ORs with 95% CIs were reported. A 2-sided *P* value < 0.05 was considered statistically significant. R (version 4.5.2) was used for statistical analysis.

## Results

### Patient cohort

The study included a total of 1,147 patients, divided into a routine angiography group from the Bad Krozingen site (n = 674) and a selective angiography group from the Freiburg site (n = 473).

As expected, the 2 groups showed notable differences in the number of performed angiography procedures post TAVI. In the routine angiography cohort, 98% (658/674) of all patients underwent postprocedural imaging, while in the selective group, only 32% (153/473) of the patients received an angiography.

The patient baseline characteristics show that the cohort in question is a typical TAVI collective (Table 1).^16^ Age, presence of cardiovascular risk factors, and an STS score of 3.2 ± 3.18 reflect a representative collective. The left ventricular function was generally good, and the valve diameters were in the middle range with a mean of 26 mm. Two-thirds of the implanted prosthetic valves were balloon-expandable valves.

**Table 1:** Baseline Characteristics of Patients Undergoing a Routine or Selective Femoral Access Post-TAVI Angiography

	Total (N = 1,147)	Selective (n = 473)	Routine (n = 674)	*P* Value
Post procedural angiography done	811 (70%)	152 (32%)	658 (98%)	***<0.001***
Age (y)	83.0 (7.0)	82.0 (7.0)	83.2 (6.4)	***0.005***
Female	555 (48%)	224 (47%)	331 (49%)	0.559
BMI (kg/m^2^)	25.9 (5.6)	25.7 (5.6)	26.1 (5.5)	***0.036***
STS score	3.20 (3.18)	4.30 (4.1)	2.70 (2.30)	***<0.001***
Cardiovascular risk factors				
Arterial hypertension	956 (83%)	378 (80%)	578 (86%)	***0.009***
Diabetes mellitus	355 (31%)	137 (29%)	218 (32%)	0.223
Hyperlipidemia/dyslipidemia	667 (58%)	281 (59%)	386 (57%)	0.470
Family history of coronary artery disease	224 (20%)	52 (11%)	172 (26%)	***<0.001***
Smoking history	207 (18%)	89 (19%)	118 (18%)	0.571
Coronary artery disease	596 (52%)	194 (41%)	402 (60%)	***<0.001***
Previous stroke	99 (8%)	83 (18%)	16 (2%)	***<0.001***
LVEF (%)	55 (9)	55 (12)	55 (9)	0.372
Aortic valve area (cm^2^)	0.8 (0.3)	0.8 (0.3)	0.8 (0.3)	0.387
Balloon-expandable prosthesis	726 (63%)	350 (74%)	376 (56%)	***<0.001***
TAVI prosthesis diameter (mm)	26.0 (3.75)	26.0 (6.0)	26.0 (3.0)	***<0.001***
Peripheral artery disease	79 (6.9%)	24 (5.1%)	55 (8.2%)	***0.042***

Baseline characteristics are shown for descriptive purposes. Covariates for multivariable adjustment were selected a priori based on clinical relevance.

Values are median (IQR) or n (%). Pearson’s Chi-squared test, Wilcoxon rank-sum test, or Fisher’s exact test as appropriate. ***Bolditalic*** numbers highlight statisticly significant values.

BMI = body mass index; LVEF = left ventricular ejection fraction; STS = Society of Thoracic Surgeons.

The baseline characteristics of both groups were largely comparable. The mean age of patients in the routine group was 83.2 years, compared to 82.0 years in the selective group. The distribution of sexes was also similar in both groups, with a slight male predominance (routine: 51%, selective: 53%). Patients in the selective group had a higher operative risk score with an STS score of 4.30 ± 4.1 than patients in the routine group (2.70 ± 2.3). Other characteristics such as arterial hypertension, diabetes, hyperlipidemia, and PAD—mostly asymptomatic—were present in both groups. The proportion of patients with a balloon-expandable TAVI valve was higher in the selective group at 74% than in the routine group at 56%. Detailed access-site characteristics are given in Table 2.

**Table 2:** Access-Site Characteristics of Patients Undergoing a Routine or Selective Femoral Access Post TAVI Angiography

	Total (N = 1,147)	Selective (n = 473)	Routine (n = 674)	*P* Value
PAD stage by Fontaine				0.122
Indefinite	35 (3.1%)	9 (1.9%)	26 (3.9%)	
I	5 (0.4%)	3 (0.6%)	2 (0.3%)	
IIa	19 (1.7%)	4 (0.8%)	15 (2.2%)	
IIb	5 (0.4%)	3 (0.6%)	2 (0.3%)	
III	1 (0.01%)	0 (0%)	1 (0.1%)	
IV	14 (1.2%)	5 (1.1%)	9 (1.3%)	
Primary access kinking				***0.041***
No/slight kinking	977 (85%)	415 (88%)	562 (83%)	
Intermediate/severe kinking	170 (15%)	58 (12%)	112 (17%)	
Primary access calcification				***<0.001***
No calcification	718 (63%)	325 (69%)	393 (58%)	
Semicircular calcification (<270°)	369 (32%)	128 (27%)	241 (36%)	
Circular calcification (>270°)	31 (3%)	14 (3%)	17 (3%)	
Stenotic calcification (<5mm diameter)	29 (2.5%)	6 (1.2%)	23 (3%)	
PAD symptoms by VRS				0.453
Asymptomatic	1,136 (99%)	468 (98%)	668 (99%)	
Mild symptoms	2 (0.2%)	0 (0%)	2 (0.3%)	
Moderate symptoms	5 (0.4%)	3 (0.6%)	2 (0.3%)	
Severe symptoms	2 (0.2%)	0 (0%)	2 (0.3%)	

Access-site characteristics are shown for descriptive purposes.

Pearson’s Chi-squared test or Fisher’s exact test as appropriate. ***Bolditalic*** numbers highlight statisticly significant values.

PAD = peripheral artery disease; TAVI = transcatheter aortic valve implantation; VRS = verbal rating scale.

### Primary outcomes: selective strategy for iliofemoral angiography after TAVI is safe

The primary outcomes of our study were bleeding complications, vascular complications, vascular reintervention, and vascular and access-related complications according to VARC-3 criteria after TAVI. Results are given in Table 3. Major and minor VARC-3 vascular and access-related complications did not show relevant differences between both strategies (6.6% and 8.0%). The proportions of bleeding complications were significantly higher in the routine group than those in the selective group (7.4% vs 16%, OR: 0.41; 95% CI: 0.27-0.61) driven by access-site bleeding (5.2% vs 13.9%, OR: 0.39; 95% CI: 0.25-0.59). Other outcomes such as closure device failure, hemoglobin drop due to any bleeding, and bleeding-related death did not show significant differences between both groups.

**Table 3:** Primary Outcomes

	Selective (n = 473)	Routine (n = 674)	OR (95% CI)	*P* Value
Vascular and access-related complications (VARC-3)	31 (6.6%)	57 (8.0%)	0.76 (0.48-1.19)	0.234
Major (VARC-3)	6 (1.3%)	12 (1.8%)	0.71 (0.25-1.84)	0.494
Minor (VARC-3)	25 (5.3%)	42 (6.2%)	0.79 (0.48-1.26)	0.321
Bleeding complications	35 (7.4%)	109 (16%)	0.41 (0.27-0.61)	***<0.001***
Access-site bleeding	28 (5.2%)	94 (13.9%)	0.39 (0.25-0.59)	***<0.001***
Closure device failure	3 (0.6%)	12 (1.8%)	0.35 (0.08-1.12)	0.107
Hemoglobin drop due to any bleeding	13 (2.7%)	26 (3.9%)	0.70 (0.35-1.36)	0.309
Death due to any bleeding	1 (0.2%)	3 (0.4%)	0.47 (0.02-3.71)	0.518
Vascular complications	40 (8.5%)	52 (7.7%)	1.11 (0.72-1.70)	0.649
Dissection	17 (3.6%)	14 (2.1%)	1.76 (0.86-3.66)	0.123
Perforation	0 (0%)	5 (0.74%)	NE	***0.004***
Stenosis	12 (2.5%)	28 (4.2%)	0.60 (0.29-1.17)	0.146
Vascular occlusion	2 (0.4%)	10 (1.4%)	0.28 (0.04-1.08)	0.103
Pseudoaneurysm	13 (2.7%)	8 (1.2%)	2.35 (0.98-5.99)	0.059
Vascular reintervention post TAVI	20 (4.2%)	44 (6.5%)	0.63 (0.36-1.07)	0.095
Surgical	8 (1.7%)	2 (0.3%)	5.76 (1.44-38.29)	***0.027***
Catheter	12 (2.5%)	42 (6.2%)	0.39 (0.19-0.73)	***0.005***
Thrombin injection	9 (2.0%)	3 (0.4%)	4.3 (1.29-19.63)	***0.028***

Four primary composite outcomes are shown along with their individual components. All outcomes were assessed using univariable logistic regression analyses and are presented as OR (95% CI). For the composite outcome, each patient is counted only once, even if multiple individual events occurred. ***Bolditalic*** numbers highlight statisticly significant values. NE indicates not estimable due to the absence of events in one group.

Vascular complications occurred in both groups in similar proportions of 7.7% and 8.5% of patients. Access-site dissection, stenosis, vascular occlusion, and pseudoaneurysm were similarly distributed in both groups, whereas access-site perforation occurred significantly more often in the routine angiography group, however with very low overall incidence (0% vs 0.74%, *P* = 0.004).

Vascular reinterventions after TAVI were slightly more frequent in the routine group (6.5%) than in the selective group (4.2%) without reaching statistical significance. Interestingly, we found significantly more surgical interventions (1.7% vs 0.3%, OR: 5.76; 95% CI: 1.44-38.29) and thrombin injections (2.0% vs 0.4%, OR: 4.3; 95% CI: 1.29-19.63) in the selective angiography group and more catheter-based interventions in the routine angiography group (2.5% vs 6.2%, OR: 0.39; 95% CI: 0.19-0.73).

In the selective group, post-TAVI angiography was performed in 5 of 8 patients (62.5%) who required surgical reintervention, in 10 of 12 patients (83.3%) undergoing catheter-based reintervention, and in 7 of 9 patients (77.7%) who required thrombin injection following TAVI.

**Figure 1:**
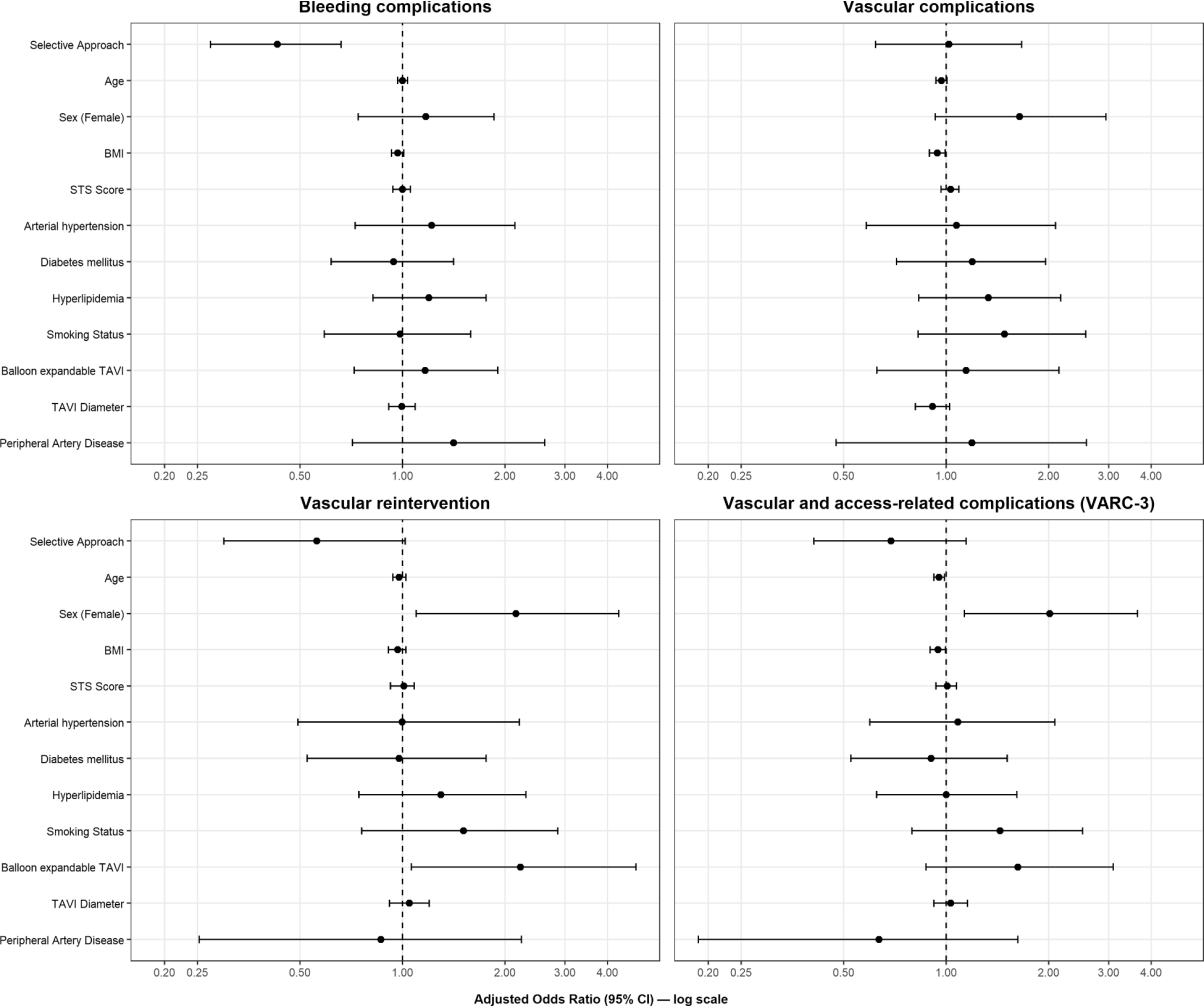
Multiple Logistic Regression Models for Primary Composite Outcomes Presented as adjusted OR with 95% CI. Significantly fewer bleeding complications occurred in the selective approach group, whereas no significant impact of the selective angiography strategy was observed on vascular complications, vascular reinterventions, or vascular and access-related complications according to VARC-3 criteria. BMI = body mass index; STS = Society of Thoracic Surgeons; TAVI = transcatheter aortic valve implantation; VARC-3 = Vascular Academic Research Consortium-3.

To adjust for potential confounding factors of major clinical relevance for our hypothesis and to determine the independent effect of each variable on the outcomes, we performed multiple logistic regression analyses. This revealed significantly lower odds of bleeding complications with the selective strategy (OR: 0.42; 95% CI: 0.29-0.61), while no additional factors were significantly associated with this outcome. There were no significant differences between the 2 strategies in the odds of VARC-3 vascular and access-related complications (OR: 0.76; 95% CI: 0.48-1.19), vascular complications (OR: 1.02; 95% CI: 0.62-1.67), or vascular reinterventions (OR: 0.56; 95% CI: 0.3-1.02). BMI was identified as a statistically significant predictor of vascular complications (OR: 0.94; 95% CI: 0.89-0.99). Female sex (OR: 2.16; 95% CI: 1.1−4.32) and ballon expandable valves (OR: 2.22; 95% CI: 1.06−4.85) were associated with an increased risk of a vascular reintervention. Female sex (OR: 2.02; 95% CI: 1.13−3.65) was also associated with VARC-3 vascular and access-related complications. The results of the logistic regression models are presented as forest plots in Figure 1.

To further strengthen the robustness of our analysis, we performed propensity score-weighted analyses. Baseline imbalances were substantially reduced, with all measured baseline covariates demonstrating adequate balance (adjusted absolute SMD < 0.10) (e.g., STS, valve type, valve diameter; all adjusted absolute SMD ≈ 0.03-0.06) (Supplemental Figures 1 and 2). The selective strategy remained associated with lower odds of bleeding complications (OR: 0.47; 95% CI: 0.29-0.75) and access-site bleeding (OR: 0.41; 95% CI: 0.24-0.67), whereas VARC-3 composite (OR: 0.66; 95% CI: 0.39-1.13), vascular complications (OR: ≈1.00; 95% CI: 0.60-1.67), and vascular reinterventions (OR: 0.58; 95% CI: 0.30-1.11) were similar (Supplemental Table 1).

Taken together, the selective angiography strategy after TAVI is associated with significantly fewer bleeding complications particularly at the access site compared to routine angiography, while rates of vascular complications and reinterventions remain comparable.

### Secondary outcomes: complications detection was not delayed through a selective strategy

The selective angiography strategy had no reducing effect on the overall fluoroscopy time.

Mean fluoroscopy time was slightly longer in the selective group than that in the routine group (median 14.4 vs 13 min; OR: 6.89; 95% CI: 2.89-16.42). Overall blood transfusions were less frequent in the selective group (6.3% vs 9.8%; OR: 0.62; 95% CI: 0.39-0.97). Detailed subgroup analyses showed no significant difference in transfusions specifically required due to access-site bleeding (Table 4).

**Table 4:** Secondary Outcomes

	Selective (n = 473)	Routine (n = 674)	OR (95% CI)	*P* Value
Blood transfusion	30 (6.3%)	66 (9.8%)	0.62 (0.39-0.97)	***0.039***
Blood transfusion due to access-site bleeding	5 (1.1%)	6 (0.9%)	1.19 (0.34-3.98)	0.784
Detection time of vascular complications				
Peri procedural	22 (4.7%)	46 (6.8%)	0.67 (0.39-1.11)	0.127
Post procedural <48 h	16 (3.4%)	15 (2.2%)	1.54 (0.75-3.17)	0.238
Post procedural >48 h	8 (1.7%)	10 (1.4%)	1.14 (0.43-2.92)	0.781
Fluoroscopy time (min)	14.43 (10.62-19.53)	13 (10-17)		***<0.001***
Iodine contrast volume (mL)	150 (120-198)	187 (145-230)		***<0.001***
Acute kidney injury	45 (9.5%)	42 (6.2%)	1.58 (1.02-2.46)	***0.041***

Secondary outcomes were assessed using univariable logistic regression analyses and are presented as OR (95% CI), except for fluoroscopy time and contrast agent volume, which were compared between groups using the Wilcoxon rank-sum test and presented with the median (interquartile range). ***Bolditalic*** numbers highlight statisticly significant values.

The timing of vascular complications did not differ significantly, indicating no relevant delay in detection of vascular complications for the selective strategy. Periprocedural, early (<48 hours), and late (>48 hours) postprocedural vascular complications show comparable rates (Table 4).

Notably, 15 of 22 patients (68%) in the selective group who experienced periprocedural vascular complications underwent post-TAVI angiography. In only one case, post-TAVI angiography was omitted despite an unrecognized primary-access occlusion, which was subsequently identified 30 minutes later based on clinical signs, prompting immediate surgical intervention. The other patients with periprocedural complications had major vascular or access-site complications (aortic dissection, major sheath-related issues, persistent bleeding), which were detected without angiography, or a clinically inapparent primary-access stenosis, which was detected before admission coincidentally. Despite similar detection-timing distributions (periprocedural, <48 hours, >48 hours), intervention modality differed: routine angiography was associated with more immediate catheter-based measures, whereas the selective strategy showed relatively more surgical and thrombin-based interventions (Table 3), indicating a shift in modality rather than delayed diagnosis.

Interestingly, acute kidney injury occurred more frequently in the selective group (9.5% vs 6.2%; OR: 1.58; 95% CI: 1.02-2.46) although significantly less iodine contrast agent was applied (150 vs 187 mL, *P* < 0.001). After stratifying for angiography status, applied contrast agent volume was lower in patients without post-TAVI angiography (170 mL vs 140 mL, *P* < 0.001) in the selective group, but acute kidney injury was distributed similarly, whereas in the routine group, no significant differences were detected (Supplemental Table 2).

**Figure 2:**
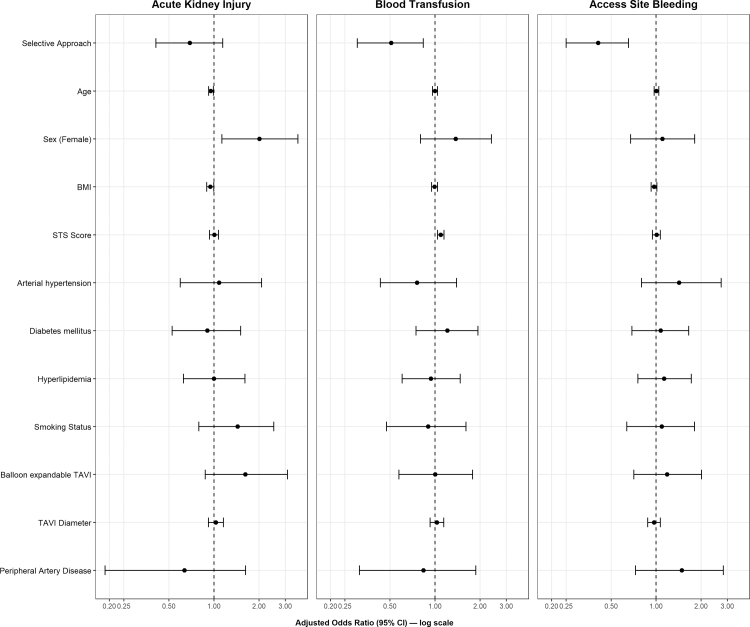
Multiple Logistic Regression Models for Secondary Outcomes Results are presented as forest plots, showing adjusted odds ratios (OR) with 95% confidence intervals (CI). Significantly lower odds of blood transfusions and access-site bleedings when using the selective angiography strategy. No significant differences on acute kidney injury between the both strategies. BMI = body mass index; STS = Society of Thoracic Surgeons; TAVI = transcatheter aortic valve implantation.

Notably, the higher rate of acute kidney injury in the selective group was observed in the univariable analysis only and was no longer significant after multivariable adjustment (OR: 1.30; 95% CI: 0.78-2.15) (Figure 2). In the propensity score–weighted model, the postprocedural angiography strategy was not associated with acute kidney injury (IPTW OR: 1.15; 95% CI: 0.68-1.97). Although total contrast volume was higher in the routine group, this did not translate into higher adjusted acute kidney injury risk. This finding suggests that the modest additional contrast required for routine iliofemoral angiography (15-25 mL) is unlikely to be clinically relevant in the context of overall TAVI contrast exposure.

**Central Illustration:**
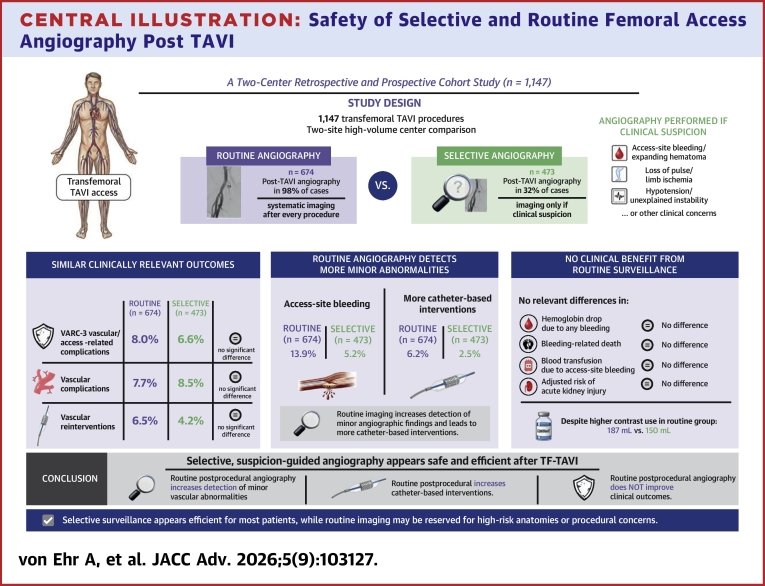
Safety of Selective and Routine Femoral Access Angiography Post TAVI A total of 1,147 TF-TAVI procedures at 2 sites of a single center with different strategies for postprocedural angiographic assessment were retrospectively analyzed. One site routinely performed iliofemoral angiography after TAVI, whereas the other applied a selective strategy based on procedural suspicion. We found that routine angiography increases detection of minor vascular abnormalities, while selective angiography guided by clinical suspicion may provide comparable clinical outcomes. TF-TAVI = transfemoral transcatheter aortic valve implantation; VARC-3 = Vascular Academic Research Consortium-3.

For the outcomes of blood transfusion and access-site bleeding, multiple logistic regression analyses were performed to adjust for the same relevant potential confounders as mentioned earlier. The results are given in Figure 2. The lower odds of blood transfusions seen in the univariable analysis persisted in the multivariable model (OR: 0.51; 95% CI: 0.30-0.84). After adjustment, the selective angiography group had a significantly lower odds of access-site bleeding (OR: 0.41; 95% CI: 0.25-0.65). Again, the propensity score–weighted model confirmed these findings (Supplemental Table 3).

## Discussion

In this high-volume 2-sites study including 1,147 patients undergoing transfemoral TAVI, we compared institutional strategies of routine vs a selective strategy for postprocedural iliofemoral angiography following transfemoral TAVI. The main findings can be summarized as follows:

First, overall vascular complication rates and the need for vascular reintervention did not significantly differ between strategies although the type of interventions differed, with more catheter-based procedures in the routine group, and more surgical interventions in the selective group. Second, routine angiography was associated with a higher detection rate of access-site bleeding and a higher rate of catheter-based vascular interventions. However, clinically relevant major and minor vascular and access-site-related complications according to VARC-3 criteria were similar between groups. Third, routine angiography was not associated with an increased risk of acute kidney injury or other procedural safety outcomes, suggesting that the kidney impact of additional contrast required for iliofemoral imaging is clinically modest (Central Illustration).

Routine postprocedural angiography increased detection of access-site abnormalities, without differences in hemoglobin drop, access-site transfusion, bleeding-related death, or VARC-3 composites. This pattern suggests overdiagnosis of minor, self-limited findings rather than preventing clinically meaningful events. A true preventive effect would typically manifest as fewer transfusions, fewer major bleeds, or fewer reinterventions—none of which were observed. The 2 strategies appear to shift intervention modality rather than outcomes. Selective, suspicion-guided imaging reduces overall interventions but accepts that a minority of events are managed later and more invasively (surgery/thrombin). Routine imaging promotes immediate catheter-based treatment of small angiographic abnormalities, increasing such procedures without measurable benefit in patient-centered outcomes. Given similar VARC-3 and vascular outcomes, selectivity seems efficient for most patients, while routine imaging can be reserved for high-risk anatomies or procedural concerns.

Taken together, these observations indicate that increased detection of angiographic abnormalities does not necessarily translate into improved clinical outcomes. Instead, routine angiography may lower the threshold for intervention when minor abnormalities are visualized, potentially resulting in treatment of findings that might otherwise resolve spontaneously. This phenomenon reflects a broader challenge in imaging-based surveillance strategies. While systematic imaging can improve detection of abnormalities, the clinical significance of these findings is not always clear. Consequently, careful interpretation of angiographic findings and judicious decision-making regarding intervention remain essential when routine imaging is employed.

Another important aspect of the present study relates to the safety of routine angiographic surveillance. One frequently cited concern regarding routine iliofemoral angiography after TAVI is the potential for increased contrast exposure and subsequent renal injury.^17^^,^^18^ Univariate acute kidney injury differences favoring the routine group despite greater contrast exposure were not sustained after multivariable adjustment or in IPTW models. This pattern supports that the modest additional contrast from routine iliofemoral angiography is unlikely to be the primary driver of acute kidney injury, whereas overall clinical risk (eg, higher STS score) and patient factors (eg, BMI) were more strongly related. Our findings therefore provide reassurance that a routine angiographic assessment does not appear to confer a measurable increase in renal risk in this cohort.

Overall vascular complication rates were similar between the 2 strategies, suggesting that selective angiographic imaging triggered by clinical suspicion is generally sufficient to identify clinically significant vascular injuries. Interestingly, however, the distribution of treatment strategies differed between groups. Catheter-based interventions were more frequent in the routine angiography cohort, whereas surgical interventions and thrombin injections were more common in the selective strategy group. These findings may reflect differences in the timing and context of complication detection. Routine angiography may facilitate earlier identification of vascular abnormalities, allowing immediate catheter-based treatment during the index procedure. In contrast, complications detected later based on clinical signs may present in a more advanced stage and therefore require surgical management. At the same time, the overall number of interventions did not differ significantly between groups, suggesting that both strategies ultimately achieve comparable clinical management of vascular complications.

Previous studies have emphasized the importance of early detection of vascular complications given their association with increased morbidity and mortality after TAVI.^19^, ^20^, ^21^ Routine angiography has been advocated to identify clinically silent vascular injuries that might otherwise remain undetected. Savvoulidis et al. recently reported that routine angiographic surveillance could reveal occult complications, potentially allowing for immediate management.^8^ However, our findings suggest that routine angiography primarily increases the detection of angiographic abnormalities, some of which may be clinically minor or self-limiting. As a result, the increased rate of catheter-based interventions in the routine angiography group may partly reflect a lower threshold for treating angiographic findings rather than a difference in clinically meaningful complications.

Importantly, the absence of differences in clinically relevant bleeding outcomes indicates that increased detection does not necessarily translate into improved patient outcomes. This observation highlights a potential challenge of imaging-based surveillance strategies: Identifying more abnormalities does not always imply clinical benefits and may lead to additional interventions whose necessity remains uncertain.

### Study limitations

While the present study provides important insights, some aspects merit consideration. First, the comparison reflects institutional practices at 2 different sites rather than a randomized comparison of 2 angiographic strategies. Differences in operator experience, procedural techniques, and institutional protocols may therefore have influenced outcomes. Although we performed multivariable adjustments for several clinically relevant variables and propensity-based weighting improved balance, residual confounding cannot be excluded. Second, the study combines retrospective and prospective data-collection periods, which may introduce differences in data completeness and event detection. Operators were not instructed regarding the study hypothesis, and data collection was blinded to analytic objectives, yet a Hawthorne effect remains possible. Third, some low-frequency outcomes (eg, perforation) limit precision. We did not have extended late follow-ups for very delayed vascular events.

## Conclusions

In this 2-site study, routine postprocedural iliofemoral angiography increased detection of minor access-site abnormalities and on-table catheter-based interventions without improving VARC-3 vascular and access-site outcomes or clinically relevant bleeding. Selective, suspicion-guided angiography achieved comparable clinical outcomes with fewer interventions. The small additional contrast from routine angiography did not translate into higher acute kidney injury risk after adjustment. Selective surveillance appears efficient for most patients, while routine imaging may be reserved for high-risk anatomies or procedural concerns. Randomized evaluations are warranted.

Perspectives **COMPETENCY IN PATIENT CARE AND PROCEDURAL SKILLS:** Routine iliofemoral angiography after transfemoral TAVI increases the detection of access-site abnormalities and is associated with more catheter-based vascular interventions. However, clinically relevant bleeding outcomes and overall vascular complication rates appear similar compared with a selective angiography strategy based on clinical suspicion.

**TRANSLATIONAL OUTLOOK:** These findings suggest that a careful clinical assessment may be sufficient in many patients, while routine angiography remains a safe option that may facilitate early detection of vascular abnormalities.

### Ethics statement

This study followed the Reporting of studies Conducted using Observational Routinely-collected health Data (RECORD) reporting guidelines. The study protocols, including both retrospective and prospective, blinded data collection and analysis, was approved by the local ethics committee (EK-Freiburg 22-1453-S1; 21-1358). Written informed consent was obtained from all patients in the prospective cohort. For the retrospective cohort, the ethics committee waived the requirement for written informed consent.

## Funding support and author disclosures

Dr Maier was funded by the IMMediate Advanced Clinician Scientist-Program, Department of Medicine II, Medical Center – University of Freiburg and Faculty of Medicine, University of Freiburg, funded by the Bundesministerium für Forschung, Technologie und Raumfahrt (BMFTR, Federal Ministry of Research, Technology and Space) - 01EO2103. Dr Heger was funded by the Berta-Ottenstein-Programme for Advanced Clinician Scientists, Faculty of Medicine, University of Freiburg. All other authors have reported that they have no relationships relevant to the contents of this study to disclose.
