# Supplementary material for: Safety of Selective and Routine Femoral Access Angiography Post-Transcatheter Aortic Valve Implantation
Source: JACC Adv. 2026 Aug 14;5(9):103127. doi: 10.1016/j.jacadv.2026.103127 (PMC13482200; doi:10.1016/j.jacadv.2026.103127)
Supplement: Supplementary file 1 [file mmc1.docx]

**Supplemental data**

| Model | Predictors | Bleeding complications | Vascular complications | Revascular intervention nach TAVI | VARC-3 |
| --- | --- | --- | --- | --- | --- |
| IPTW-weighted | ClinicFR | 0.47 (0.29–0.75) | 1.00 (0.60–1.67) | 0.58 (0.30–1.11) | 0.66 (0.39–1.13) |

**Supplemental Table S1.**

**Supplemental Table S2. Contrast volume and incidence of acute kidney injury stratified by angiography status.** Continuous variables are presented as median with interquartile range and categorical variables as number (%). Pearson’s Chi-squared test or Wilcoxon rank sum test as appropriate.

|  | **selective (n=473)** | | **p** | **routine (n=674)** | | **p** |
| --- | --- | --- | --- | --- | --- | --- |
|  | angio done (n=153) | no angio (n=320) |  | angio done (n=658) | no angio (n=16) |  |
| contrast volume (ml) | 170 (129 - 230) | 140 (110 – 180) | <0.001 | 187 (145 – 230) | 173.5 (148 – 216) | 0.794 |
| Acute kidney injury | 19 (12.4%) | 26 (8.1%) | 0.137 | 41 (6.2%) | 1 (6.3%) | 0.998 |

**Supplemental Table S3**

| Model | Predictors | Acute Kidney Injury | Blood Transfusion | Access Site Bleed |
| --- | --- | --- | --- | --- |
| IPTW-weighted | ClinicFR | 1.15 (0.68–1.97) | 0.59 (0.34–1.02) | 0.41 (0.25–0.67) |

**Supplemental Figure S1.**

**Supplemental Figure S2.** Love plot illustrating covariate balance before and after inverse probability of treatment weighting (IPTW) based on the propensity score model used for sensitivity analysis. Standardized mean differences (SMDs) for baseline covariates are shown for the unweighted cohort and after IPTW adjustment. Covariate balance was considered adequate at an absolute SMD <0.1. The IPTW procedure substantially reduced baseline imbalances between groups, indicating improved comparability after weighting.
